# Preparation of an Antibacterial Branched Polyamide 6 via Hydrolytic Ring-Opening Co-Polymerization of ε-Caprolactam and Lysine Derivative

**DOI:** 10.3390/polym16141997

**Published:** 2024-07-12

**Authors:** Xiaoyu Mao, Wei Liu, Zeyang Li, Shan Mei, Baoning Zong

**Affiliations:** 1 Research Center of Renewable Energy, Research Institute of Petroleum Progressing, SINOPEC, Beijing 100083, China; maoxiaoyu.ripp@sinopec.com (X.M.); liuwei2.ripp@sinopec.com (W.L.); lizeyang.ripp@sinopec.com (Z.L.); 2 NO. 22 Research Department, Research Institute of Petroleum Progressing, SINOPEC, Beijing 100083, China; meishan.ripp@sinopec.com; 3 State Key Laboratory of catalytic Material and Reaction Engineering, Research Institute of Petroleum Progressing, SINOPEC, 18th Xueyuan Road, Haidian District, Beijing 100083, China

**Keywords:** antibacterial PA6, branched PA6, hydrolysis open-ring polymerization, thermal properties, rheological properties, mechanical properties, antibacterial activity

## Abstract

In this study, we successfully realized the hydrolytic ring-opening co-polymerization of ε-caprolactam (CPL) and lysine derivative. A novel antibacterial modified polyamide 6 with a branched structure was obtained after the quaternization of the co-polymers. The co-polymers exhibited a significant increase in zero shear viscosity, melt index and storage modulus at the low frequency region. The quaternized co-polymers displayed thermal properties different from pure PA6 and good mechanical (tensile) properties. The antibacterial activity of the quaternized co-polymers depends on the quaternary ammonium groups’ incorporated content. At 6.2 mol% incorporation of quaternary ammonium groups, the strong antibacterial activity has been introduced to the co-polymers. As the quaternary ammonium groups approached 10.1 mol%, the antibacterial polymers demonstrated nearly complete killing of *Staphylococcus aureus* (Gram positive) and *Escherichia coli* (Gram negative). The above research results provided a new approach for the study of high-performance nylon.

## 1. Introduction

Polyamide 6 (PA6), also known as Nylon 6, is a kind of versatile polymer with a wide range of applications. It is primarily used in fibers (80–90%), engineering plastics, adhesives, and packing materials [1,2,3,4]. Since first obtained, PA6 as well as its monomer ε-caprolactam (CPL) has resulted in remarkable development in the synthesis technology, with PA6 now accounting for over 80% of the total nylon production [5]. Given the continuous expansion of PA6 production capacity and the growing demand for high-performance materials, the functional modification of PA6 has become a key development focus. Especially in recent years, the COVID-19 pandemic has heightened public awareness of hygiene and health, making the study of antibacterial materials a current research hot spot [6,7]. With biocompatibility, excellent mechanical properties and high resistance to organic solvents, oils, and bodily fluids, PA6 is suitable for the materials of medical equipment [8,9]. However, it is inherently non-antibacterial. Thus, the development of antibacterial PA6 through the incorporation of functional components is crucial. 

Antibacterial PA6 can be prepared via modification techniques and post-treatment techniques [10]. Adding antibacterial agents during the polymerization or spinning process is considered the main method of modification techniques, while coating or impregnating PA6 with antibacterial components is involved in post-treatment techniques. Antibacterial PA6 prepared through modification techniques exhibits long-lasting antibacterial effects, but the other properties of the polymer may be impacted with the introduction of antibacterial components.

Erem et al. [11] prepared antibacterial PA6 fibers with different silver nanoparticle (AgNP) contents using the melt intercalation method. Antibacterial activity tests showed that the fibers exhibited high bactericidal rates against *Staphylococcus aureus* (Gram positive) and *Klebsiella pneumoniae* (Gram negative), and the bactericidal rates against both were enhanced with the increasing silver content. However, the partial aggregation of AgNP components led to the generation of defects in PA6, resulting in a decrease in the tensile properties of antibacterial PA6 fibers prepared by this method. In addition, the high thermal conductivity of AgNP components accelerate the cooling of the polymer melt, resulting in a decrease in the crystallinity. Buchenska et al. [12] grafted acrylic acid (AA) onto PA6 and combined antibiotics with the introduced carboxyl groups to create antibacterial PA6 fibers, which demonstrated good antibacterial properties against both Gram-positive and Gram-negative bacteria. Shi et al. [13] grafted chitosan onto PA6 molecular chains under the initiation of potassium persulfate. Although the grafting rate was low (1.58 mol%), the grafted PA6 showed a bactericidal rate of 89% against Gram *Staphylococcus aureus* and over 90% against *Escherichia coli* and *Candida albicans*. In addition, there was no significant decrease in the bactericidal rate of the modified antibacterial PA6 against the aforementioned bacteria after 50 washes. Zhang et al. [14] employed co-irradiation to graft poly(methacrylic acid-2-(dimethylamino) ethyl ester) (PDMAEMA) onto PA66, followed by protonation and anion exchange, resulting in antibacterial PA66 fibers. These fibers exhibited strong antibacterial efficacy against *Candida albicans* and *Staphylococcus aureus*, with a slight decrease in mechanical properties. Lian et al. [15] obtained the co-polymer of CPL and dimethyl-protected cyclic lysine (DMCL) mediated by t-BuP_4_, followed by quaternization with 1-bromoethane. When the quaternized ammonium groups content reached 17 mol%, the product exhibited a 99% bactericidal rate against *Staphylococcus aureus* and *Escherichia coli*, albeit with some reduction in thermal and mechanical properties. 

**Figure 1:**
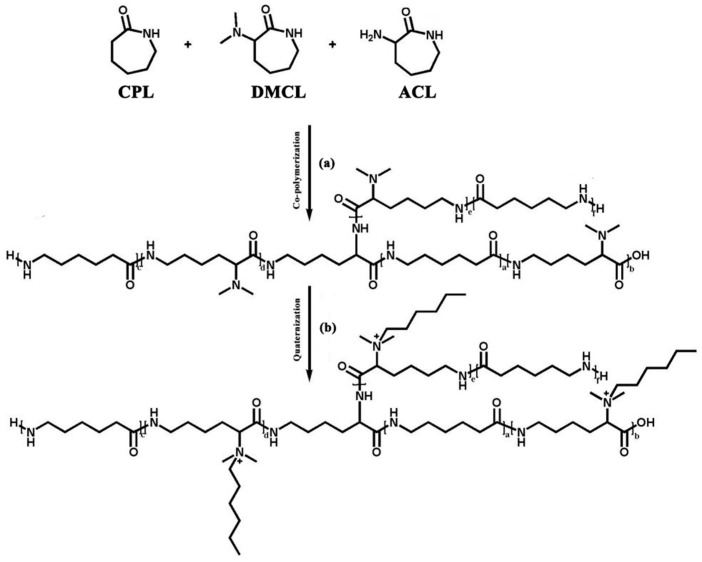
The main reaction of this system.

Based on the above research results, the introduction of antibacterial groups may have an impact on the thermal and mechanical properties of polymers. However, the above two properties play an important role in the processing and use of polymers. Therefore, it is necessary to prepare an antibacterial polymer with excellent thermal and mechanical properties. In the previous research of our research group, a branched co-polymer was synthesized by introducing α-Amino-ε-caprolactam (ACL) into the CPL polymerization process [16]. When the amount of ACL added was appropriate, the thermal properties of the co-polymer had little change compared to pure PA6, and the tensile properties were significantly improved, and the rheological properties showed obvious change. In addition, our research group has previously achieved hydrolytic ring-opening co-polymerization of CPL and DMCL. Based on the above achievements, ACL was introduced to prepare a co-polymer with a branched chain structure during the co-polymerization of CPL and DMCL (Figure 1a), and the resulting product was subjected to rheological properties and tensile properties tests. Then the co-polymer underwent quaternization with 1-Bromohexane (Figure 1b). The thermal properties as well as the antibacterial activities against *Staphylococcus aureus* and *Escherichia coli* of the co-polymer were tested.

## 2. Materials and Methods

### 2.1. Materials

The CPL used in this study was supplied by Baling Petrochemical Company, SINOPEC (Yueyang, China). ACL as well as DMCL was synthesized in our earlier work. Sulfuric acid and 2,2,2-Trifluoroethanol was purchased from Acros (Fukuoka, Japan). 1-Bromohexane provided by Innochem (Beijing, China) was used to be the quaternization reagent in our research.

### 2.2. Co-Polymerization

The reactant mixture consisted of CPL, DMCL and ACL, with the initiator, deionized water, and these were added to a reactor. Vacuuming was applied to remove the O_2_ component in the reactor and then N_2_ gas was inflated to form the N_2_ atmosphere. The pre-polymerization was carried out at 225 °C, lasting for 1 h, followed by 6 h polymerization at 250 °C under pressure. Then, the reactant mixture was co-polymerized under atmospheric pressure for 1 h, followed by another 2 h reaction below −0.05 MPa. The obtained co-polymers were discharged and cut into pellets. The residual monomers and low molecular weight polymers contained in theco-polymers pellets were removed through the water bath extraction at 90 °C.

### 2.3. Quaternization of the Co-Polymers

The mixed liquid consisted of the co-polymers 2,2,2-Trifluoroethanol solution and the quaternized reagent 1-romohexane and was kept at 70 °C for a 24 h reaction. Then the solvent and unreacted quaternized reagent were removed through the rotary evaporation. The quaternized co-polymer samples were obtained after stoving.

### 2.4. Characterization and Test of Properties

The capillary viscometer was used to determine the relative viscosity (*η*_r_), intrinsic viscosity ([*η*]), and viscosity-average molecular weight (*M_W_*) of the co-polymers. The co-polymers were dissolved in a 96 wt% sulfuric acid solution (solute:solvent, 1 g:100 mL). The time it took for the solvent (*t*_0_) and the co-polymers solution (*t*) to pass through the capillary at 25 °C was measured. The above-mentioned feature can be calculated through the following equations.

$$Relative\;viscosity\;(\eta_{r})\;=\frac{t}{t_{0}}$$

$$Specific\;viscosity\;(\eta_{sp})\;=\eta_{r}-1$$

$$\mathrm{Intrinsic}\;viscosity\;([\eta ])\;=\frac{\sqrt{2({\;\eta }_{sp}-ln\eta_{r})}}{c}$$

the *M_w_* can be obtained through the Mark–Houwink equation:

[*η*] = *k* [*M_w_*]^a^.

The Mark–Houwink constants for PA6 in a 25 °C, 96% sulfuric acid solution are as follows [17]:

*k* = 6.3 × 10^−4^ *a* = 0.764

The structures of the co-polymers were investigated by ^1^H NMR (AVANCE NEO 500 M, Bruker, Billerica, MA, USA), using formic acid/trifluoroacetic acid-d (1:1, v:v) as the solvent. To check the rheological properties of co-polymers, a rotational rheometer (P25CSL, HAAKE MARS, Waltham, MA, USA) was used. The samples were prepared by injection molding at 240 °C in a mold with a 20 mm diameter and 1 mm thickness. Complex viscosity (η), storage modulus (G′) and loss modulus (G″) of the co-polymers were tested through a frequency sweep ranging from 0.1 to 500 rad/s under oscillatory mode with a strain of 1%. The melt index was tested by the melt index instrument (MI40, GOETTFRT, Essen, Germany). The test temperature was 230 °C, and the load was 2.16 kg. The mass of the sample passing through the capillary column for a certain period of time was recorded. 

The thermal properties of the co-polymers were determined by differential scanning calorimetry (DSC3, METLER TOLEDO, Zurich, Switzerland) and thermal gravimetric analysis (RT-800, METLER TOLEDO, Zurich, Switzerland) under N_2_ atmosphere. In DSC, the samples were first heated from 25 °C to 300 °C to remove the thermal history, with the rate of 10 °C/min. Then, the samples were cooled to 25 °C and heated to 300 °C again at the sample rate. The degree of crystallinity (Xc) can be obtained by the following equation.

*X*_c_ = Δ*H*_m_/Δ*H*_0_ × 100%

where Δ*H*_m_ is the specific enthalpy of melting, and Δ*H*_0_ is the enthalpy of melting with 100% crystalline PA6 (188 J/g) [18].

The tensile mechanical properties were checked by the universal tensile machine (CMT2000, MTS/SANS, Eden Prairie, MN, USA) according to ASTM D638 [19] with the strain rate of 10 mm/min. All tested co-polymer samples were dried for 24 h in a desiccator under vacuum. 

### 2.5. Antimicrobial Activities Test of Co-Polymers after Quaternization

The surface antibacterial activities of quaternized co-polymers were tested according to GB/T31402-2015 [20]. Culture medium, liquid culture medium, and test samples were prepared before the test as well as the activation of *Staphylococcus aureus* (Gram positive) and *Escherichia coli* (Gram negative). The activated two types of bacteria were inoculated onto liquid culture medium, followed by incubation at 36 °C for 24 h, and then, the bacterial suspension can be obtained after diluting. An appropriate amount of bacterial suspension was added onto the surface of PA6 samples and the quaternized co-polymers samples to be tested. Then the bacterial suspension was covered with polypropylene film and squeezed evenly. After a 24 h incubation at 36 °C, the bacterial suspension was retrieved. Then it was applied to the surface of the culture dish after dilution, followed by incubation at 36 °C for 24 h. The number of colonies in the culture dish was calculated by the plate counting method. The antibacterial activities were determined by comparing the number of colonies in the PA6 samples and the quaternized co-polymers samples.

## 3. Results

### 3.1. The Reaction Mechanism of Co-Polymerization

**Figure 2:**
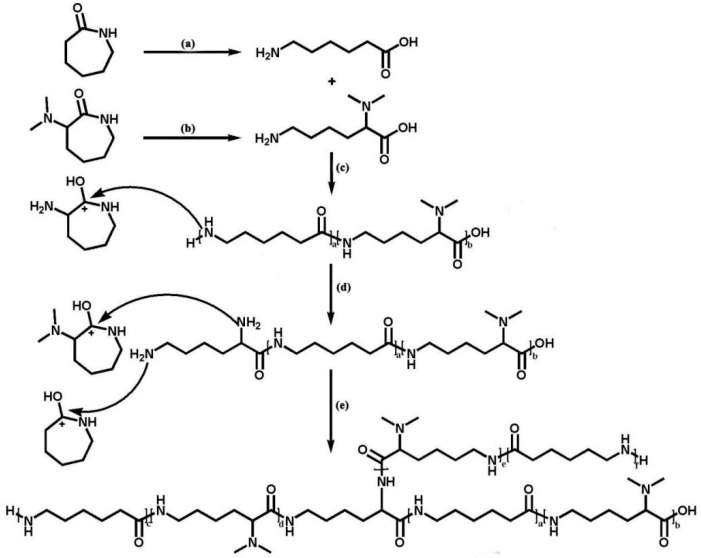
The reaction mechanism of CPL and ACL: (**a**) The ring-opening of CPL. (**b**) The ring-opening of DMCL. (**c**) The poly-condensation of linear monomers. (**d**) The incorporation of two chain growth sites. (**e**) The growth of the main chain and the side chain.

The reaction mechanism of our co-polymerization system is similar to the synthesis of PA6 via hydrolytic ring-opening polymerization of CPL. Under high temperature and with water existence, the amide bonds of the monomers (CPL and DMCL) break, resulting in the formation of 6-aminocaproic acid and linear DMCL (Figure 2a,b). Low molecular weight co-polymers are generated through poly-condensation between linear monomers (Figure 2c). The terminal amino groups of the low weight co-polymers attack the protonated ACL, which leads the branching agent added to the co-polymers chain (Figure 2d). The incorporation of ACL contributes to the two chain growth sites in the co-polymers. The two amino groups in a co-polymer chain can attack the protonated monomers, through which the growth of the main chain and the side chain can be realized (Figure 2e). A kind of branched co-polymer is obtained through the above reaction steps.

### 3.2. The Structure Characterization of the Co-Polymers

**Figure 3:**
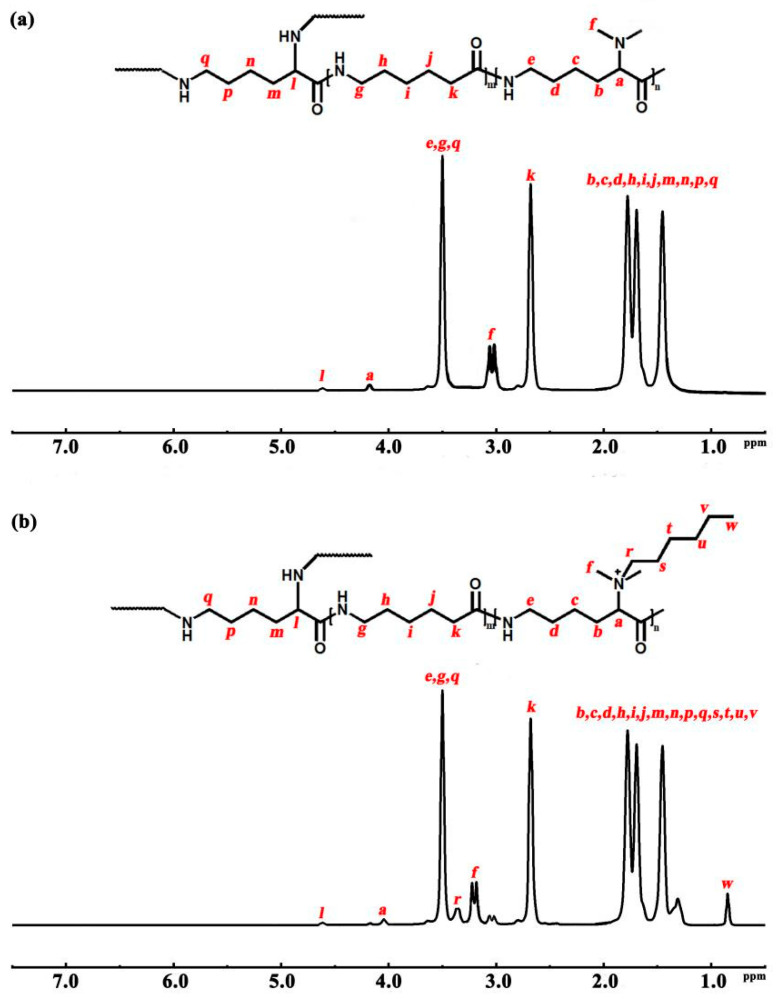
The ^1^H NMR of the co-polymers (**a**) and the quaternized co-polymers (**b**).

The structure of the co-polymers and the quaternized co-polymers were determined by ^1^H NMR. The structural formula and ^1^H NMR spectrum of the co-polymers are shown in Figure 3a, with the chemical shifts of H atoms as follows: δ4.67 (*l*, branching site); δ4.18 (a, dimethylamino site); δ3.48, 2.68, 1.42–1.75 (main chain and side chain); δ3.12 and δ3.05 (f, methyl H). The structural formula and ^1^H NMR spectrum of the quaternized co-polymers are given in Figure 3b, with the chemical shifts of H atoms as follows: δ4.67 (*l*, branching site); δ4.07 (a, dimethylamino site); δ3.48, 2.68, 1.42–1.75 (main chain, side chain and quaternized groups); δ3.25 and δ3.18 (f, methyl H); δ3.33 and δ0.82 (quaternized groups). 

### 3.3. Monomer Conversion, Configuration and Incorporation of the Co-Polymers

**Figure 4:**
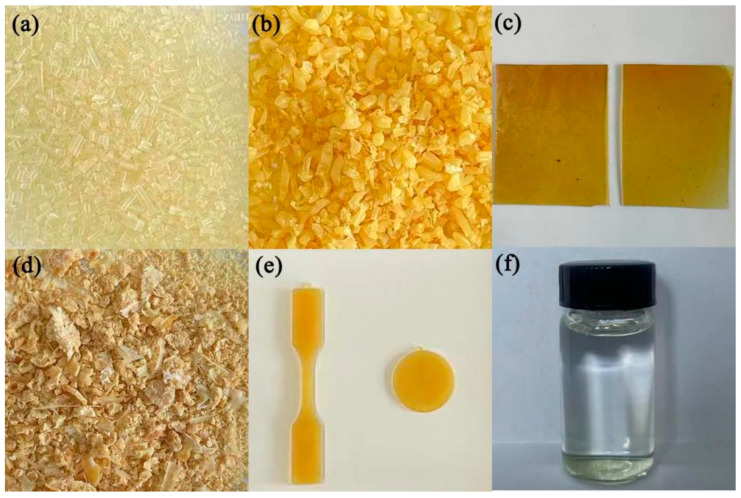
(**a**–**c**) The co-polymers with the feed shown in entry 2, 5 and 7 in Table 1, respectively. (**d**) The quaternized co-polymer with the feed shown in entry 5 in Table 1. (**e**) The samples after molding. (**f**) The co-polymers solution.

The co-polymers obtained under different feed ratios are shown in Figure 4a–c. When the proportion of DMCL in the feed was below 22 mol%, the co-polymers can be drawn and pelletized after extrusion. The PA6 pellets, shown in Figure 4a, indicated that the melt strength of the co-polymers was sufficiently high to maintain continuity during drawing. When the DMCL content in the feed was 30 mol%, the melt strength of the co-polymers failed to meet the requirements for drawing and pelletizing, and the pellets were obtained by simple crushing (Figure 4b). When the DMCL content in the feed reached 50 mol%, the co-polymers obtained after cooling exhibited properties similar to elastomers, suggesting that the co-polymers had converted to an amorphous state (Figure 4c). When the DMCL content in the feed reached 70 mol%, the co-polymer appeared as a gel rather than a solid at room temperature, and it had a certain solubility in hot water. Table 1 shows that with a constant feed amount of ACL, the relative viscosity and molecular weight of the co-polymers decreased with the increase in the DMCL proportion in the feed. This corresponded with the aforementioned morphological changes of the co-polymers. The monomers in the feed cannot be completely co-polymerized into the molecular chain. As shown in Table 1, there was little change in the conversion of ACL and CPL, while the conversion of DMCL was significantly affected by its content in the feed. It is due to the notably lower polymerization activity of DMCL compared to CPL, that the polymerization rate is influenced by the reactivity and monomers concentration. When the concentration of DMCL was low, its polymerization rate was considerably smaller than that of CPL, leaving a substantial amount of un-polymerized DMCL at the end of the reaction. In the feed ratio shown in entry 11, the proportion of DMCL to CPL in the co-polymer molecular chains approaches 1:1, indicating that the sample was just like an alternating co-polymer.

### 3.4. Rheological Properties Test of Co-Polymers with Different ACL Feed

**Figure 5:**
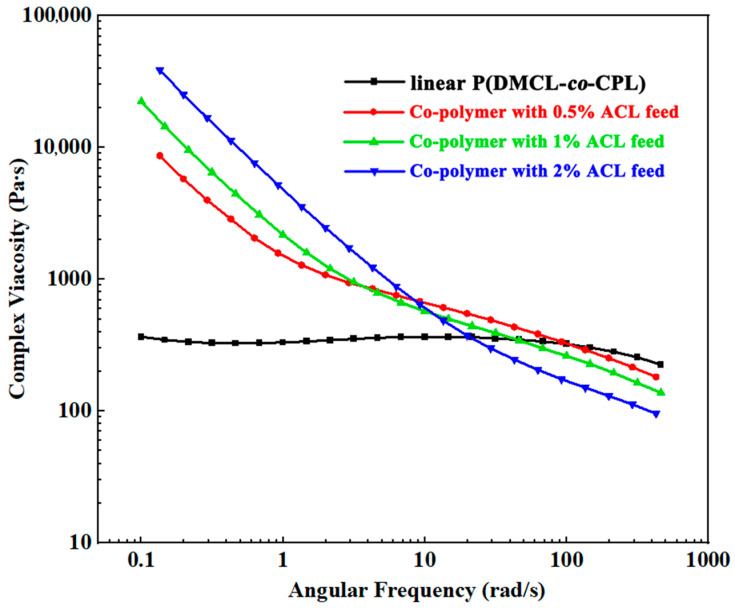
The complex viscosity of the co-polymers.

It has been proven that the rheological properties of polymers are closely related to the molecular chain structure [21]. During the co-polymerization process, incorporating different amounts of ACL can generate different amounts of branched chains, thereby altering the rheological properties of the co-polymers. In this section, co-polymers with different ACL feed content were prepared while keeping the DMCL feed content constant. The feed ratio and rheological characteristic parameters of the co-polymers are shown in Table 2. Figure 5 exhibits the complex viscosity (*η*) of co-polymers with different AACL feed melt as a function of angular frequency (*ω*). The Newtonian liquid behavior was observed through the *η-ω* curve of linear P(DMCL-*co*-CPL). A Newtonian plateau of low viscosity appeared at low frequency. As the ACL was incorporated, the complex viscosity of the melt increased substantially higher than that of linear P(DMCL-*co*-CPL). Meanwhile, the Newtonian plateau of these samples disappeared, and the strong shear-thinning behavior was observed. As the incorporated DMCL increased, the shear thinning behavior became more obvious. The above phenomenon was caused by the formation of the branched structure and the entanglement effect of molecular chains. According to the mechanism of co-polymerization mentioned in Figure 2, the generation of the branched structure was formed with the incorporation of ACL, followed by the intensified entanglement effect between molecular chains. The movement of the chain was hindered by the increasing entanglement effect, which contributed to shearing and deformation not being instantaneous, manifested externally as the increase in melt viscosity at low frequency and exhibiting non-Newtonian fluid behavior [22]. The zero-shear viscosity is the complex viscosity of the melt when the shear rate tends to zero and the system approaches an equilibrium state. It can be calculated through the simple Carreau equation with the Cox–Merz rule [23]

η(γ)/η_0_ = (1 + (γτ_n_)^2^)^(n−1)/2^

where η_0_ is the zero-shear viscosity, γ is the shear rate, τ_n_ is the characteristic time, and n is a parameter. It is notable in Table 2 that as the is ACL incorporated, the zero-shear viscosity increased more than 50 times. For linear chain polymers, the increase in *M_w_* can also enhance the zero-shear viscosity. The 3.4 power-law is used to describe the relationship of zero shear viscosity and *M_w_* [24]. However, the increase in molecular weight of Sample 1 and Sample 2 can only result in a five-fold increase in the zero-shear viscosity, assuming that they follow the 3.4 power-law. For Sample 3, the zero-shear viscosity increased as well as a decreasing *M_w_*, which was unable to follow the law. The above phenomena indicate that changes in molecular weight are not the main factor affecting zero-shear viscosity. The huge increase in zero-shear viscosity can be also explained with the generation of gel structures after chemical cross-linking [22]. Cross-linked polymers can only swell in a solution without dissolving. Furthermore, as thermosetting materials, cross-linked polymers cannot be hot worked after molding [25]. However, the co-polymers we obtained can easily dissolve in 2,2,2-Trifluoroethanol (Figure 4f) and be transformed into a test sample through a molding injection (Figure 4e). Thus, we believed that there are no cross-linked structures in our co-polymers.

It can be also observed that as the ACL was incorporated, the melt index exhibits an increase. During the testing process of the melt index, the shear rate is high and the entanglement between the molecular chains is released, leading to the disappearance of the effect of the viscosity increase caused by branched chains disappearance. As the amount of ACL incorporated increases, the branched chain content increases, and the hydrogen bonds between the co-polymer molecular chains are disrupted. The macroscopic manifestation is that the resistance to internal flow of the co-polymer melt is weakened, the flow performance of the co-polymer improves, and the melt index increases. This is consistent with the variation pattern of the complex viscosity of the co-polymers at high frequency in Figure 5.

**Figure 6:**
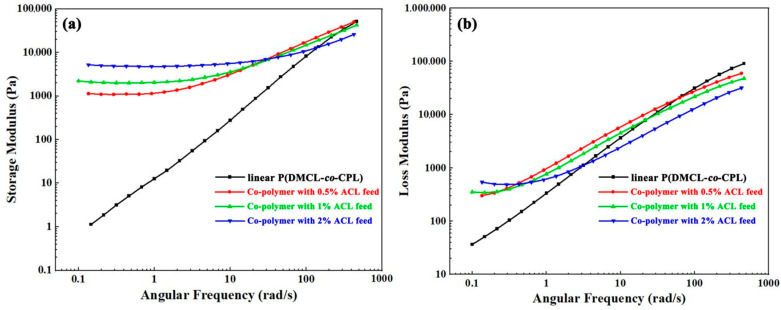
The storage modulus (**a**) and loss modulus (**b**) of the co-polymers.

Figure 6a,b show the variation of storage modulus (G′) and loss modulus (G″) with the angular frequency (ω), respectively. The storage modulus is the elastic, solid-like behavior, while the loss modulus is the viscous response [26]. The melt of linear polymers exhibits typical terminal behaviors at the low frequency region. The G′–ω curve and G″–ω are close to straight lines, whose slopes are 2 and 1, respectively, in logarithmic coordinates [27]. The G′–ω curve of P(DMCL-*co*-CPL) exhibited like a straight line with a slope close to 2 in the low-frequency region, agrees with the terminal behaviors. As the introduction of ACL, the G′–ω curve deviated significantly from the terminal behavior. More branched chains strengthened the entanglement of the molecular chains, with enhancement of the solid-like behavior of the co-polymer melt and prolongation of the relaxation time. At the low frequency region, the decrease in G′ with ω is not significant. Meanwhile, the enhancement of solid-like behavior means that the energy stored in the melt increases during an alternating stress cycle, corresponding to a nearly 1000-fold increase in the storage modulus of ACL-incorporated co-polymers over P(DMCL-*co*-CPL) at the low frequency region.

### 3.5. Mechanical Properties of the Co-Polymers

**Figure 7:**
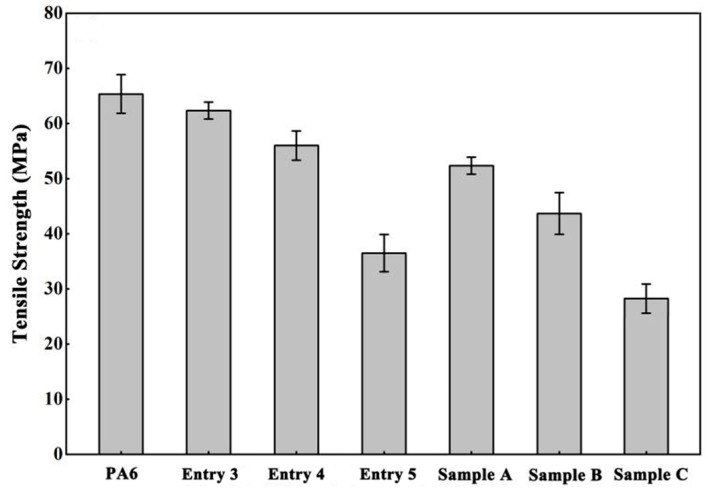
The mechanical properties of the PA6 and co-polymers.

The mechanical properties of the PA6 and the co-polymers(Entry 3–5 in Table 1) are shown in Figure 7. Linear DMCL/CPL co-polymers with the same content of DMCL (Sample A-C) were the control. Samples A-C have the same DMCL incorporated as Enrty 3–5, respectively. The linear co-polymers exhibit a significant decrease in tensile strength compared to pure PA6; this is because the introduction of DMCL significantly reduces the crystallinity of the polymer. For the co-polymers of Entry 3 and Entry 4, despite the reduction of crystallinity, the tensile strength was improved with the ACL incorporated because the molecular weight as well as the branches had increased, and more chain entanglement was proceeded. During the tensile process, each entanglement site of the chain needs to be disentangled after reaching the yield, resulting in an increase in the total stress required, which is reflected in the enhancement of tensile strength. For the co-polymer of Entry 5, due to a significant decrease in crystallinity, its tensile strength is lower than PA6.

### 3.6. Quaternization of the Co-Polymers

The solid co-polymers (Entry 2–7 in Table 1) were quaternized by reacting with 1-bromohexane, and the quaternized products were sequentially named as Samples 1–6. The quaternization rate was determined by the ^1^H NMR, and the content of quaternary ammonium groups incorporated was calculated, as shown in Table 3. It can be seen that in the quaternization reaction of the co-polymer with 1-bromobutane, the quaternization rate remained around 80%.

### 3.7. Thermal Properties Test of Co-Polymers with Different Quaternary Ammonium Groups Incorporated

**Figure 8:**
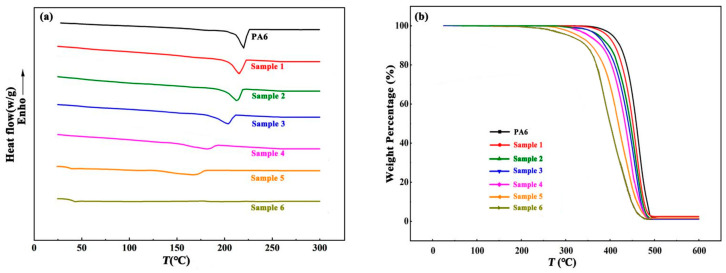
(**a**) DSC scanning curve and (**b**) TG curve of PA6 and Samples 1–6.

Figure 8a shows the DSC scanning curve obtained from the second heat progress of PA6 and Samples 1–6 with different incorporated quaternary ammonium groups. The thermal properties parameters are shown in Table 4. PA6 exhibited a sharp melting peak at 221.2 °C. As the quaternary ammonium groups incorporated increased, the melting peak became wide and moved to a low-temperature area, which was reflected in polymer properties as a decrease in melting point and crystallinity. When the incorporated quaternary ammonium groups reached 16.8 mol% (Sample 5), the melting peak became particularly wide, and the glass transition temperature was observed on the curve. At the highest level of quaternary ammonium group incorporation (30.2 mol%, Sample 6), the melting peak disappeared, which meant that the co-polymer was amorphous and there was no crystallization. Short-branched chains, formed during the branching process, as well as the quaternary ammonium groups affects, the arrangement of the main chain and long branch chains, hindering their formation of ordered and regular structures [27]. As the main chain gradually forms an ordered arrangement, the areas where short chains gather form amorphous regions. The appearance of amorphous regions affects the overall structural regularity of the polymer, leading to a decrease in its melting point and crystallinity. Compared to pure PA6, the thermal stability of co-polymers decreases. All of the co-monomers, branched chains, and quaternary ammonium groups had an impact on the regularity of the molecular chains, disrupting the regularity, resulting in abatement in hydrogen bonding and intermolecular forces, leading to the decrease in thermal stability [28]. 

### 3.8. Antibacterial Activities Test of Co-Polymers

**Figure 9:**
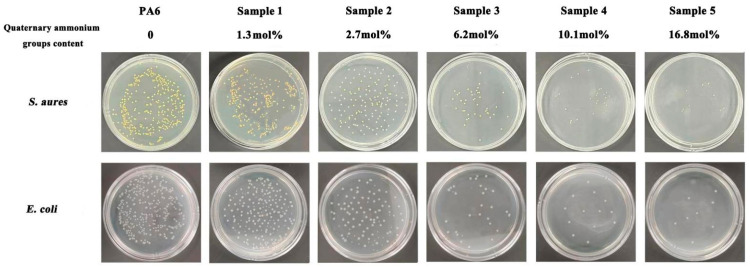
The results of antimicrobial activities test.

Quaternized co-polymers with crystallinity and capable of being molded into sample pieces were selected for the antibacterial activities test. The results are shown in Figure 9. The small yellow or white dots on the culture dish represent the surviving bacterial colonies. Due to the absence of antibacterial activities, the bacterial content in the bacterial suspension did not decrease after a 24 h culture on the PA6 sample piece. Therefore, when the bacterial suspension is recovered, diluted, and transferred to a culture dish for another 24 h, dense colonies of bacteria can be obviously seen. It has been proved that long chain PA6 containing quaternary ammonium groups has antibacterial activities. Quaternary ammonium groups have a positive charge, while bacterial cell membranes composed of phospholipid bilayers have a negative charge. When the two come into contact, the quaternary amine groups will adsorb onto the bacterial cell membrane due to the electrostatic interactions, inhibiting bacterial growth and division. In addition, the long chains of aliphatic polyamides have good compatibility with phospholipids. Long molecular chains can penetrate the cell membrane, destroy the cell membrane skeleton, and cause bacterial cell membrane rupture [29]. After the bacterial suspension underwent a 24 h culture on the piece of the co-polymer with 6.2 mol% quaternary ammonium group incorporation (Sample 3), a significant decrease in colonies appeared on the culture dish. The antibacterial rate of this co-polymer sample against *Staphylococcus aureus* and *Escherichia coli* was calculated by the plate counting method to be around 85%. When the incorporated quaternary ammonium groups reached 10.1 mol% (Sample 4 and 5), the antibacterial rate of the co-polymer sample against the above two bacteria reached 99.9%, indicating that the co-polymer demonstrated nearly complete killing of *Staphylococcus aureus* and *Escherichia coli*.

## 4. Conclusions

In this study, we successfully synthesized an antibacterial branched PA6 with CPL, ACL and DMCL via hydrolytic ring-opening co-polymerization, followed by the quaternization with 1-Bromohexane. The met index (MFR), zero-shear rate viscosity and storage modulus at the low frequency region of the co-polymers have a remarkable increase as well as the shear thinning phenomenon becoming more obvious. The melting point, degree of crystallinity and thermal stability of the quaternized co-polymers decreases with the increase in functional monomers incorporated. The quaternized co-polymers with 6.2 mol% quaternary ammonium groups incorporated (Sample 3) exhibit excellent antimicrobial activities against *Staphylococcus aureus* (Gram positive) and *Escherichia coli* (Gram negative) and when the quaternary ammonium groups incorporated reach 10.1% (Sample 4), the co-polymers demonstrate complete killing of the two above bacterium. This research provides a new method for the preparation of high performance modified PA6.

## Figures and Tables

**Table 1:** Monomer conversion and incorporation of the co-polymers.

Entry	Feed ACL/DMCL/CPL	*η_r_*	*M_w_*	Conversion(%) ACL/DMCL/CPL	Incorporation ACL/DMCL/CPL
1	0/0/100	2.63	17010	0/91.2	-
2	1/4/95	2.51	15850	35.7/33.4/92.1	0.4/1.6/98
3	1/8/91	2.49	15640	35.3/36.2/91.1	0.4/3.4/96.2
4	1/13/86	2.42	14910	35.9/41.2/91.2	0.5/7.4/92.1
5	1/22/77	2.20	12610	34.8/47.3/90.9	0.4/12.8/86.8
6	1/30/69	1.98	10290	33.7/54.1/90.2	0.4/20.7/78.9
7	1/50/49	1.89	9340	34.1/63.2/91.4	0.4/37.4/62.2
8	1/70/29	1.42	4410	33.1/75.1/90.2	0.3/49.9/49.8

**Table 2:** Rheological properties of the co-polymers with different ACL feed.

Entry	Feed ACL/DMCL/CPL	*η_r_*	*M_w_*	Zero Shear Viscosity (Pa·s)	Melt Index
1	0/13/87	2.31	13780	405	71.4
2	0.5/13/86.5	2.40	14470	20542	251
3	1/13/86	2.42	14910	31454	279
4	2/13/85	2.38	14510	49523	386

**Table 3:** Quaternization rate and quaternary ammonium group content.

Sample	Feed ACL/DMCL/CPL	Incorporation DMCL Content	Quaternization Rate (%)	Quaternary Ammonium Groups Incorporated (%)
1	1/4/95	1.6	80.2	1.3
2	1/8/91	3.4	80.9	2.7
3	1/15/84	7.4	81.9	6.2
4	1/22/77	12.8	79.7	10.1
5	1/30/69	20.7	81.2	16.8
6	1/50/49	37.4	80.1	30.2

**Table 4:** The thermal properties of PA6 and Samples 1–6.

The Polymer	Quaternary Ammonium Groups Incorporated (%)	Melting Point (°C)	Crystallinity (%)	Initial Thermal Degradation Temperature (°C)	Maximum-Rate Thermal Degradation Temperature (°C)
PA6	0	221.2	27.38	424.4	458.1
Sample 1	1.3	211.4	25.59	414.2	456.2
Sample 2	2.7	209.8	24.26	401.2	453.1
Sample 3	6.2	201.4	22.32	398.4	451.9
Sample 4	10.1	189.2	20.19	382.2	450.7
Sample 5	16.8	168.4	18.20	361.1	449.2
Sample 6	30.2	-	-	283.4	398.6

## Data Availability

Data are contained within the article.
